# Phytolacca for Cockroaches

**Published:** 1874-09

**Authors:** 


Dr. T. C. Renner writes to the Department of Agriculture that several years ago he collected some poke-root (*Phytolacca decandra*) for medicinal purposes, and spread it at several places about the house to dry. Soon afterward he observed that there were many cockroaches lying dead, and upon examination found that they had been partaking freely of the poke-root. Some of the root was placed near their haunts, and the result was that it rid the premises of those insects. Since then he has communicated the remedy to others, who have tested it with satisfactory results.

